# N-acetylaspartate from fat cells regulates postprandial body temperature

**DOI:** 10.21203/rs.3.rs-3835159/v1

**Published:** 2024-01-09

**Authors:** Jessica B. Felix, Pradip K. Saha, Evelyn de Groot, Lin Tan, Robert Sharp, Elizabeth S. Anaya, Yafang Li, Holly Quang, Nooshin Saidi, Layla Abushamat, Christie M. Ballantyne, Christopher I. Amos, Philip L. Lorenzi, Samuel Klein, Xia Gao, Sean M. Hartig

**Affiliations:** 1 Division of Diabetes, Endocrinology, and Metabolism, Baylor College of Medicine, Houston, TX; 2 Department of Medicine, Baylor College of Medicine, Houston, TX; 3 Department of Molecular and Cellular Biology, Baylor College of Medicine, Houston, TX; 4 Cancer and Cellular Biology Program, Baylor College of Medicine, Houston, TX; 5 Metabolomics Core Facility, Department of Bioinformatics and Computational Biology, The University of Texas MD Anderson Cancer Center, Houston, TX; 6 Institute for Clinical and Translational Research, Baylor College of Medicine, Houston, TX; 7 Section of Epidemiology and Population Sciences, Department of Medicine, Baylor College of Medicine, Houston, TX; 8 Children’s Nutrition Research Center, Department of Pediatrics, Baylor College of Medicine; 9 Data Sciences Program, Whiting School of Engineering, Johns Hopkins University, Baltimore, MD; 10 Cardiovascular Research, Department of Medicine, Baylor College of Medicine, Houston, TX; 11 Center for Human Nutrition, Washington University School of Medicine, St. Louis, MO

## Abstract

N-acetylaspartate (NAA), the brain’s second most abundant metabolite, provides essential substrates for myelination through its hydrolysis. However, activities and physiological roles of NAA in other tissues remain unknown. Here, we show aspartoacylase (ASPA) expression in white adipose tissue (WAT) governs systemic NAA levels for postprandial body temperature regulation. Proteomics and mass spectrometry revealed NAA accumulation in WAT of *Aspa* knockout mice stimulated the pentose phosphate pathway and pyrimidine production. Stable isotope tracing confirmed higher incorporation of glucose-derived carbon into pyrimidine metabolites in *Aspa* knockout cells. Additionally, serum NAA positively correlates with the pyrimidine intermediate orotidine and this relationship predicted lower body mass index in humans. Using whole-body and tissue-specific knockout mouse models, we demonstrate that fat cells provided plasma NAA and suppressed postprandial body temperature elevation. Furthermore, exogenous NAA supplementation reduced body temperature. Our study unveils WAT-derived NAA as an endocrine regulator of postprandial body temperature and physiological homeostasis.

White adipose tissue (WAT) is primarily known for its role as an energy reservoir, storing and releasing energy depending on whole-body nutritional demands. WAT safely sequesters large quantities of excess nutrients as lipids in fat cells. In contrast, the accumulation of excess lipids in other tissues drives insulin resistance^1^. WAT also serves as a major endocrine organ by secreting bioactive proteins, small molecules, and lipids that work locally and systemically to help coordinate energy balance^2^. Importantly, endocrine interactions among nerve fibers and adipocytes regulate fat mass by communicating signals for metabolic homeostasis, which are involved in the regulation of vital physiological functions, including hunger, body temperature, and glucose homeostasis^3^. Perturbations in WAT functions often appear in metabolic disorders, including obesity, emphasizing the importance of understanding how fat cells perform precise sensing and regulation of energy balance.

Metabolites produced within AT or by other endocrine organs provide signaling molecules that can directly impact AT function and whole-body metabolism. These signaling metabolites activate^4^ or inhibit glycolysis^5^, supply substrates for post-translational modifications^5,6^, promote lipid burning^4^, and regulate inflammatory pathways in the surrounding WAT microenvironment^4,5^. Some metabolites also affect food intake and glucose homeostasis by regulating the directed release of WAT adipokines^7^. Although a few efforts have defined small molecules accumulating within AT that act on peripheral metabolism, the functional relationships between metabolite abundance and physiological outcomes remain poorly defined.

The metabolite N-acetylaspartate (NAA) is found in high quantities in the brain, second only to the neurotransmitter glutamate^8^. Despite being discovered over six decades ago, NAA function and its link to various diseases remains largely uncharacterized^8^. One of its suggested roles is that of acetate carrier from neurons to the surrounding oligodendrocytes, where the hydrolytic enzyme aspartoacylase (ASPA) is expressed^8^. ASPA catalyzes the deacylation of NAA to regenerate cytoplasmic aspartate and supply acetate for the acetyl-CoA units required for myelin lipid synthesis. Consistent with this activity, loss-of-function mutations in *Aspa* cause Canavan disease, a lethal condition associated with NAA accumulation and degeneration of myelin lipids in the brain^9^. Here, we discovered that WAT expresses the machinery to synthesize and degrade NAA. When NAA levels were systemically elevated using *Aspa* knockout approaches, we observed a greater abundance of pyrimidine precursors in plasma and WAT that lowered fat mass. Moreover, our studies reveal a previously uncharacterized role for NAA and ASPA in the regulation of pyrimidine biosynthesis in WAT and downstream regulation of postprandial body temperature.

## Results

### *Aspa* is abundantly expressed in WAT and induced during adipocyte differentiation.

NAA is the only ASPA substrate, and its hydrolysis supplies acetate crucial for lipid synthesis in oligodendrocytes, the myelin-forming cells of the central nervous system. However, expression analysis in the Genotype-Tissue Expression (GTEx) Portal and the Human Protein Atlas identified abundant ASPA levels in tissues outside the brain. We found that *Aspa* mRNA levels were more than five-fold greater in mouse adipose tissues relative to whole brain. In contrast, other metabolic tissues did not show meaningful changes in gene expression (Extended Data Fig. 1a). This observation may derive from predicted binding sites for the pro-adipogenic transcription factor peroxisome proliferator-activated receptor gamma (PPARγ) in the proximal promoter region of ASPA that regulates gene expression in mouse and human adipocytes^10–12^. We tested this prediction by measuring *Aspa* gene expression during adipocyte differentiation in three *in vitro* human and mouse models. Similar to known mature adipocyte marker genes (*Adipoq*, *Fabp4*, *Pparg2*), *Aspa* gene expression was increased in mouse adipocytes differentiated from stromal vascular fraction (SVF) and 3T3-L1 cells (Extended Data Fig. 1b–c). Human adipocytes also showed robust induction of *ASPA* and confirmed broad shared expression patterns across species (Extended Data Fig. 1d). ASPA protein levels were also higher in mature adipocytes (Extended Data Fig. 1e) and exhibited diffuse localization patterns throughout the cell (Extended Data Fig. 1f). Finally, we used liquid chromatography-mass spectrometry (LC-MS) to assess metabolite levels in 3T3-L1 preadipocytes and differentiated adipocytes. NAA levels increased in mature adipocytes, along with most TCA cycle metabolites and the anaplerotic metabolite glutamate (Extended Data Fig. 1g). These findings demonstrate that ASPA expression is uniquely enriched in adipose tissue. Additionally, NAA and ASPA levels respond to the signals that promote adipocyte differentiation.

### *Aspa* knockout decreases body weight and raises metabolic flexibility in mice.

**Fig. 1 |:**
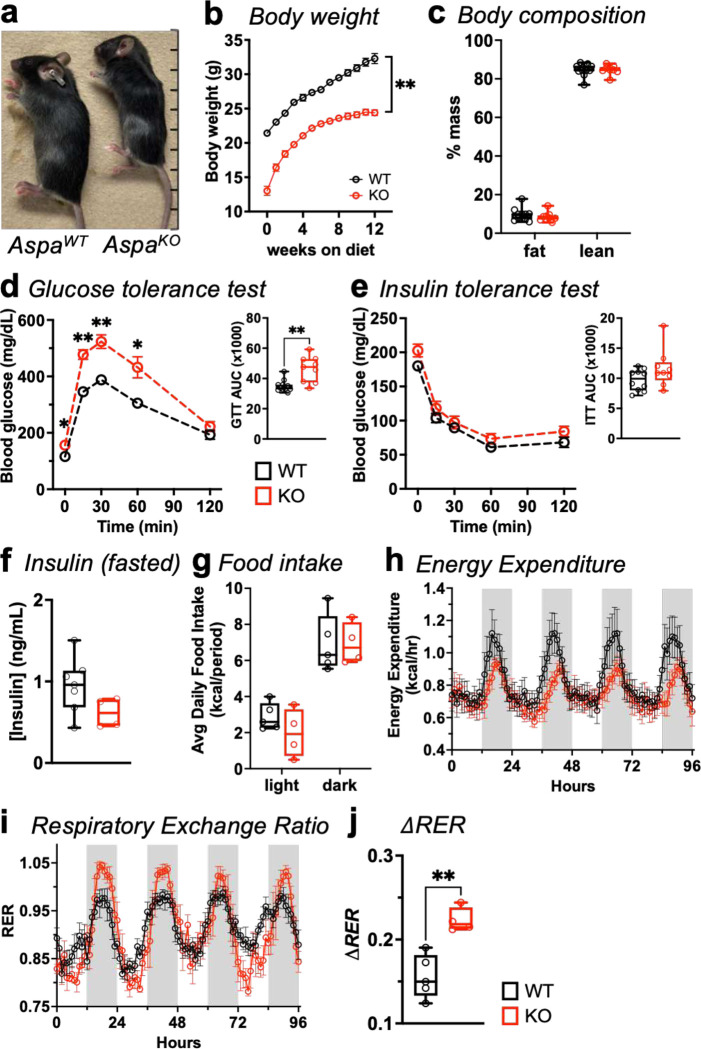
Constitutive *Aspa* deletion significantly affects body weight and substrate utilization in mice fed normal chow diet. *Aspa*^*WT*^ and *Aspa*^*KO*^ mice were fed normal chow diet (NCD) for 12 weeks followed by metabolic phenotyping. **a,** Visual representation of *Aspa*^*WT*^ and *Aspa*^*KO*^ mice at 8 weeks of age. **b,** Body weight measurements of *Aspa*^*WT*^ and *Aspa*^*KO*^ mice over 12-week period starting at 6 weeks of age (n=6,10 mice/group). Data represent mean ± s.e.m. **P<0.01 by ordinary two-way ANOVA. **c,** Body composition by Echo MRI shown as percentage of body weight (n=11,9 mice/group). **d,** Glucose tolerance test (GTT) and **(e)** insulin tolerance test (ITT) with corresponding area-under-curve (AUC) measurements. Data represent mean ± s.e.m. *P<0.05, **P<0.01 by mixed-effects model followed by Sidak’s multiple comparisons test. AUC: **P<0.01 by unpaired two-tailed Student’s *t*-test. **f,** Serum insulin levels following 16 h fast (n=7,4 mice/group). Mice were individually housed and monitored in CLAMS cages during a 96 h period with measurements of: **(g)** food intake, **(h)** energy expenditure (EE), and **(i)** respiratory energy ratio (RER) (n=5,4 mice/group). Data represent mean ± s.e.m. Statistical analysis of **(g-i)** was performed by ANCOVA with lean body mass as a covariate. **j,** delta RER (*Δ*RER), defined as the difference between the average of the lowest 10% (light) and highest 10% (dark) of RER values (n=5,4 mice/group). **P<0.01 by unpaired two-tailed Student’s *t*-test. **d-e,** AUC**, c,f,g,j,** Data represented as box-and-whisker plots using the Min-to-Max method in GraphPad Prism: box limits, 25^th^ to 75^th^ percentiles; center line, median; whiskers, minimum and maximum values.

To explore how ASPA contributes to energy balance, we performed phenotyping of whole-body *Aspa* knockout mice (*Aspa*^*KO*^)^13^. *Aspa*^*KO*^ were born at a normal Mendelian ratio and were viable, but male mice failed to grow (Fig. 1a) and gain weight (Fig. 1b) during the first 12 weeks of life. Body composition studies established the genotypes accumulated similar percentages of fat and lean weight when normalized to body weight (Fig. 1c). In addition to the expected central nervous system phenotypes, the Knockout Mouse Project and International Mouse Phenotyping Consortium (IMPC; https://www.mousephenotype.org/^)14^ identified fasting hyperglycemia in *Aspa*^*KO*^ lines. Similarly, glucose tolerance tests (GTT) and corresponding area-under-the-curve calculations showed *Aspa*^*KO*^ mice cleared glucose more slowly compared to *Aspa*^*WT*^ mice (Fig. 1d). Despite glucose intolerance, insulin tolerance (Fig. 1e) and fasted serum insulin levels (Fig. 1f) were similar across groups.

Higher body mass predicts higher total energy expenditure^15,16^. To characterize the effect of whole-body *Aspa* deletion on energy balance, mice were housed in metabolic cages for five days and data were analyzed for genotype-specific effects in CalR^17^. Despite the lower body weight and smaller size of *Aspa*^*KO*^, food intake did not vary across genotypes (Fig. 1g). While *Aspa*^*KO*^ mice trended towards lower energy expenditure compared to *Aspa*^*WT*^ mice, ANCOVA suggested any difference between the groups occurred primarily due to body weight, not genotype (Fig. 1h).

Respiratory exchange ratio (RER) reflects whole-body substrate preference, where shifts from low RER to high RER during light-to-dark cycles indicate fatty acid versus carbohydrate oxidation, respectively. While total energy expenditure was not different among genotypes, *Aspa*^*KO*^ mice exhibited higher RER during the dark phase (Fig. 1i). The difference between the light phase (lower RER) and dark phase (higher RER) reflects the metabolic flexibility and adaptation to altered fuel availability^18^. Based on RER amplitude changes, *Aspa*^*KO*^ mice deftly switch fuel sources and achieve greater metabolic flexibility during light-to-dark transition (Fig. 1j).

### *Aspa* knockout results in smaller adipocytes and greater abundance of pyrimidines.

**Fig. 2 |:**
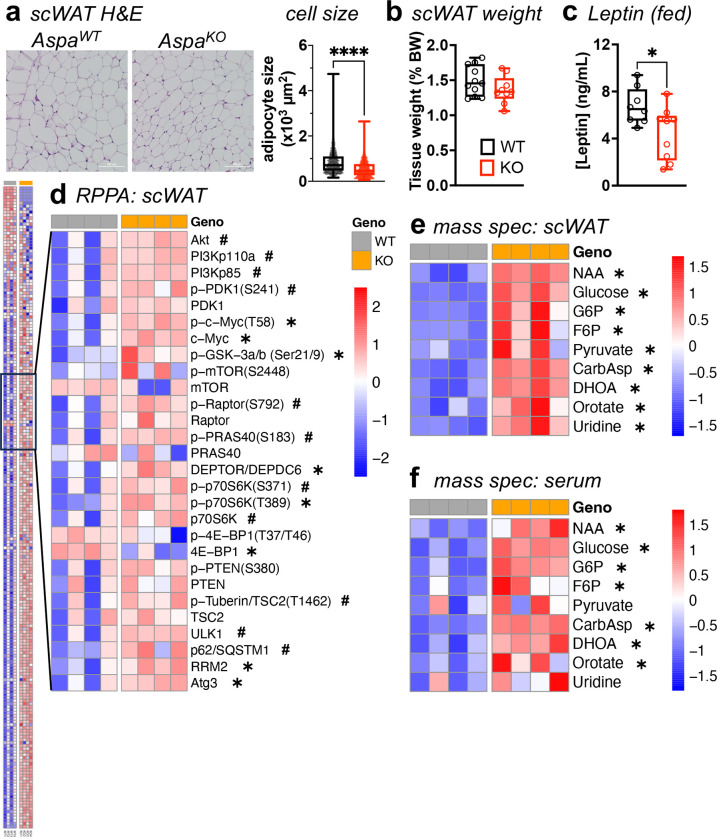
*Aspa* deletion leads to smaller adipocytes that produce pyrimidine metabolites. scWAT from *Aspa*^*WT*^ and *Aspa*^*KO*^ mice fed NCD for 12 weeks were analyzed for tissue morphometry and protein expression. **a,** Representative histological analysis by Hematoxylin & Eosin (H&E) and mean adipocyte cell size (μm^2^) of scWAT across 3–5 fields of view (n=4 mice/group); scale bars, 100μm. **b,** scWAT depot tissue weight, shown as percentage of body weight (n=11,9 mice/group). **c,** Fed serum leptin levels (n=8,9 mice/group). **a-c,** Data represented as box-and-whisker plots using the Min-to-Max method in GraphPad Prism: box limits, 25^th^ to 75^th^ percentiles; center line, median; whiskers, minimum and maximum values. **d,** Reverse-phase protein array (RPPA) analysis of scWAT from *Aspa*^*WT*^ and *Aspa*^*KO*^ mice. Relative abundance of NAA, glycolytic, and pyrimidine metabolites in **(e)** scWAT and **(f)** serum of *Aspa*^*WT*^ and *Aspa*^*KO*^ mice, measured by IC/MS targeted analysis. **d-f,** Data shown as z-score of log_2_ transformed values (n=4 mice/group). **a-f,** ^*#*^*P*<0.1, *P<0.05, ****P<0.0001 by unpaired two-tailed Student’s *t*-test.

Based on the observation that ASPA increased during adipocyte differentiation and the pro-adipogenic effects of the other proximal pathway lipogenic enzymes ACSS2 and ACLY^6,19,20^, we hypothesized *Aspa* depletion would generate less competent fat cells. To test this hypothesis, we collected subcutaneous WAT (scWAT) and visceral WAT (vWAT) depots from *Aspa*^*KO*^ mice and littermate controls for immunohistochemistry and molecular analysis. While adipocytes were smaller in scWAT (Fig. 2a) and vWAT (Extended Data Fig. 2a), scWAT weight was unchanged across genotypes when normalized to body weight (Fig. 2b). vWAT weight was lower in *Aspa*^*KO*^ mice even after normalization to body weight (Extended Data Fig. 2b). In agreement with reduced WAT mass, ad-libitum serum leptin levels were lower in *Aspa*^*KO*^ mice (Fig. 2c).

To characterize cell signaling events underpinning smaller adipocyte size and metabolic flexibility in *Aspa*^*KO*^ , we applied the WAT samples to a reverse-phase protein array (RPPA) with broad pathway coverage of 240 antibody probes (Fig. 2d and Extended Fig. 2c). *Aspa*^*KO*^ tissues showed altered activity of proteins involved in various pathways, including insulin signaling (Akt), protein synthesis (4EBP1), and cell proliferation (Myc). More specifically, we found *Aspa*^*KO*^ enriched for the mTOR pathway in WAT, including increased levels of phospho-p70S6K (T389), and components of the mTORC1 complex (Raptor, Deptor). In parallel, we leveraged ion chromatography-mass spectrometry (IC-MS) to screen for metabolite concentrations in adipose tissue depots and serum of *Aspa*^*WT*^ and *Aspa*^*KO*^ mice. As expected, NAA was higher in the scWAT and vWAT of *Aspa*^*KO*^ mice (Fig. 2e, Extended Data Fig. 2d). We also found higher abundance of glucose, pyruvate, and the pyrimidine metabolites carbamoyl aspartate (CarbAsp), dihydroorotate (DHOA), orotate, and uridine in WAT of *Aspa*^*KO*^ mice compared to controls. Serum metabolites showed similar accumulation of metabolites altered in WAT from *Aspa*^*KO*^ mice, including NAA and pyrimidine intermediates (CarbAsp, DHOA, and orotate) (Fig. 2f). Taken together, the combination of RPPA and metabolomics in WAT identified that NAA elevation from *Aspa* knockout promoted de novo pyrimidine synthesis that is supported by higher activity of anabolic and nutrient-sensing pathways.

### NAA correlates with pyrimidine abundance in human serum.

**Fig. 3 |:**
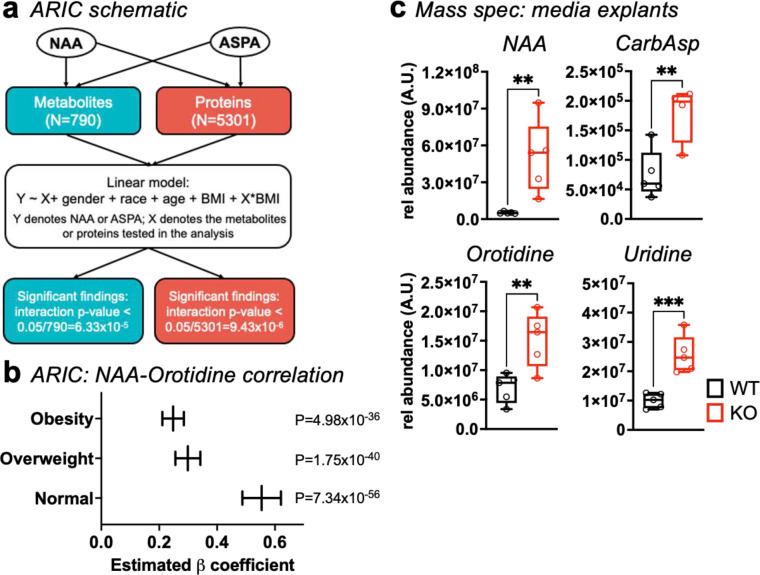
ARIC reveals NAA and the pyrimidine metabolite oritidine predicts body composition. **a,** Schematic for analysis of Atherosclerosis Risk in Communities (ARIC) dataset using an age-, sex-, race-, and BMI-adjusted linear regression model. **b,** Estimated effect of orotidine on NAA (β coefficient) for each BMI category, along with respective 95% confidence intervals and statistical significance from linear regression model. **c,** Relative abundance of NAA and pyrimidine metabolites in media from scWAT explants incubated 24 h. Data represented as box-and-whisker plots using the Min-to-Max method in GraphPad Prism: box limits, 25^th^ to 75^th^ percentiles; center line, median; whiskers, minimum and maximum values. **P<0.01, ***P<0.001 by unpaired two-tailed Student’s *t*-test.

Serum NAA accumulates in the rare inherited disorder Canavan disease, but relationships among more common conditions remain poorly investigated. The Atherosclerosis Risk in Communities (ARIC) is a large-scale, longitudinal study that measures associations of serum risk factors of heart disease^21^. To explore potential interactions of NAA and ASPA with other proteins and metabolites in humans, we performed linear correlation analysis using mass spectrometry data from ARIC adjusted for gender, race, age, and BMI (Fig. 3a). Among the participants in ARIC, we observed a significant association between NAA and the pyrimidine intermediate orotidine across BMI categories, and these correlations decreased with obesity (Fig. 3b). We did not find any metabolites or proteins that correlated with circulating ASPA.

The greater abundance of pyrimidine metabolites in *Aspa*^*KO*^ serum and WAT aligned with ARIC analysis showing that NAA positively and uniquely correlated with orotidine. Based on these findings, we predicted that ASPA governs NAA secretion from WAT for systemic effects. To explore how ASPA contributes to NAA release from WAT, we incubated scWAT explants from *Aspa*^*WT*^ and *Aspa*^*KO*^ mice in DMEM followed by mass spectrometry analysis of the media to assess differences in metabolite secretion. We found that NAA was highly abundant in the media of *Aspa*^*KO*^ and far higher than levels observed in explants from *Aspa*^*WT*^, indicating that NAA made in WAT is secreted into the surrounding microenvironment (Fig. 3c). We also detected higher levels of CarbAsp, orotidine, and uridine in the media from *Aspa*^*KO*^ mice, strengthening the notion that elevated NAA regulates pyrimidine synthesis in WAT.

### NAA accumulation in *Aspa* knockout adipocytes increases CAD activity.

**Fig. 4 |:**
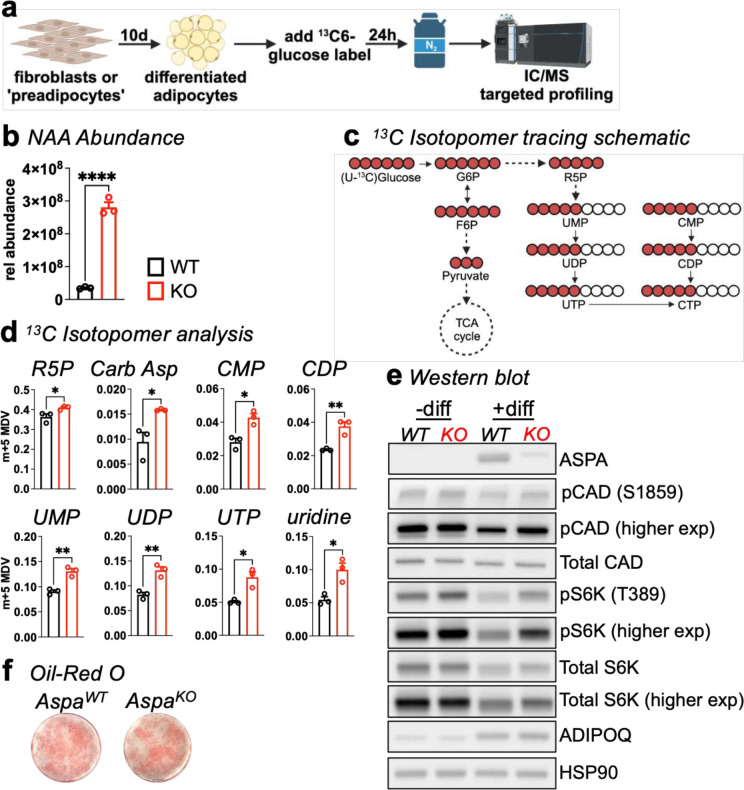
*Aspa*^*−/−*^ adipocytes shunt glucose-derived carbon into pyrimidines. **a,** Schematic of targeted metabolomic profiling of SVF-derived differentiated adipocytes isolated from *Aspa*^*WT*^ and *Aspa*^*KO*^ mice. Glucose incorporation into metabolite pools was measured by incubating cells with U^13^C glucose. Isotopolog distribution for indicated metabolites was measured after 24 h using IC-MS targeted profiling. **b,** Relative abundance of whole-cell NAA (n=3 replicates/group). Data represent mean ± s.e.m. ****P<0.0001 by unpaired two-tailed Student’s *t*-test. **c,** Schematic showing contribution of ^13^C_6_-labeled glucose tracing into ribose-5-phosphate and related pyrimidine metabolites by looking at m+5 isotopologs. **d,** Fractional labeling of R5P and pyrimidine metabolites. Isotopolog data are corrected for ^13^C natural abundance (n=3 replicates/group). Data are mean ± s.e.m. *P<0.05, **P<0.01, ****P<0.0001 by unpaired two-tailed Student’s *t*-test. **e,** Immunoblot of *Aspa*^*+/+*^ and *Aspa*^*−/−*^ primary SVF cells ± differentiation, probed for phosphor-CAD, total CAD, phospho-S6K, total S6K, and ADIPOQ. HSP90 served as the loading control. **f,** Representative Oil-Red O (ORO) staining of lipid droplets in differentiated *Aspa*^*WT*^ and *Aspa*^*KO*^ SVF adipocytes (n=3 replicates/group).

Glucose supplies most of the carbon for biosynthetic reactions in adipocytes^22^. Our data imply NAA accrual in WAT directs the prioritized flux of carbon into pyrimidine synthesis. To directly assess pyrimidine synthesis in our models, we performed stable isotope tracer analysis using uniformly-labelled ^13^C6 glucose in SVF-derived adipocytes, followed by targeted profiling of metabolites by IC-MS (Fig. 4a). As expected, NAA abundance was eight-fold higher in *Aspa*^*KO*^ cells compared to controls (Fig. 4b). To explore ^13^C assimilation into pyrimidines, we focused on m+5 isotopologs derived from the pentose phosphate pathway product ribose-5-phosphate (Fig. 4c). Incorporation of the label into ribose-5-phosphate (R5P) and pyrimidine metabolites (CarbAsp, CMP, CDP, UMP, UDP, UTP, and uridine) was greater in *Aspa*^*KO*^ cells, indicating that this unopposed increase in NAA forced glucose flux through the pentose phosphate pathway and pyrimidines (Fig. 4d).

We also examined protein expression in SVF cells from *Aspa*^*WT*^ and *Aspa*^*KO*^ mice before and after adipocyte differentiation. As expected, ASPA was not present in preadipocytes, highly expressed in differentiated *Aspa*^*WT*^ cells, and absent in *Aspa*^*KO*^ cells (Fig. 4e). Mechanistically, mTORC1 activation of p70S6K allows phosphorylation of carbamoyl phosphate synthetase II, aspartate transcarbamylase, and dihydroorotase (CAD) at Ser1859^23^ to catalyze the first three steps in de novo pyrimidine synthesis. In line with this idea, *Aspa*^*KO*^ results in sustained p70S6K and CAD phosphorylation in adipocytes (+diff). While *Aspa*^*KO*^ cells selectively engage in pyrimidine synthesis, adipocyte differentiation was nominally higher as measured by the adipocyte marker ADIPOQ (Fig. 4e) and Oil-Red O staining of neutral lipids (Fig. 4f). Together, our data support a critical role for ASPA in regulating pyrimidine metabolism in adipocytes.

### ASPA expression regulates postprandial body temperature.

**Fig. 5 |:**
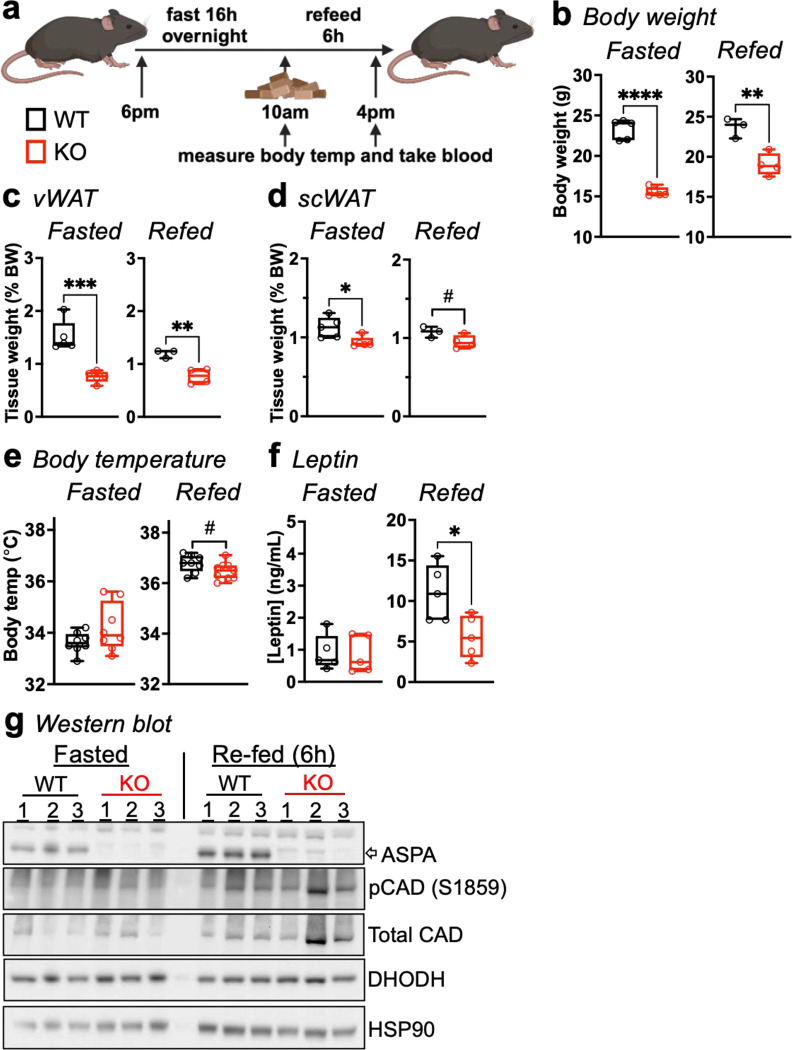
ASPA expression governs postprandial body temperature. **a,** Schematic of fast/re-feeding experiment. Body temperature measurements and serum of *Aspa*^*+/+*^ and *Aspa*^*−/−*^ mice were taken following 16 h fast and after 6 h of refeeding. Necropsy data from fasted and refed mice: **(b)** Body weight, **(c)** gWAT and **(d)** scWAT depot weights, shown as % of body weight. Fasted (n=5 mice/group), refed (n=3,4 mice/group). **e,** Body temperature measured by rectal probe (n=8–9 mice/group). **f,** Serum leptin levels (n=5 mice/group). **g,** Immunoblot of scWAT from *Aspa*^*+/+*^ and *Aspa*^*−/−*^ mice following fasting and re-feeding, probed for p-CAD, total CAD, p-S6K, total S6K, DHODH, ASPA (n=3 mice/group/condition). **(b-f)** Data represented as box-and-whisker plots using the Min-to-Max method in GraphPad Prism: box limits, 25^th^ to 75^th^ percentiles; center line, median; whiskers, minimum and maximum values. ^#^*P*<0.1, *P<0.05, **P<0.01, ***P<0.001, ****P<0.0001 by unpaired two-tailed Student’s *t*-test.

Adipocyte secretion of pyrimidines during fasting regulates postprandial body temperature^24^. Our studies demonstrated that *Aspa* deletion increases systemic pyrimidines, and the correlation between NAA and *de novo* pyrimidine synthesis in the cellular models likewise suggested a regulatory relationship that impacts regulation of postprandial body temperature. To examine whether heightened NAA and pyrimidine levels from *Aspa* knockout affected body temperature, we fasted *Aspa*^*WT*^ and *Aspa*^*KO*^ mice overnight for 16 h, after which we measured rectal body temperature and collected blood from the tail vein (Fig. 5a). Mice were then refed for six h followed by another round of body temperature measurement and blood collection. In both the fasted and re-fed states, *Aspa*^*KO*^ mice weighed 7–10 g less than *Aspa*^*WT*^ mice (Fig. 5b) and showed depleted vWAT and scWAT mass relative to body weight (Fig. 5c,d). In line with the idea that NAA promotes a functional pool of pyrimidines, we found that body temperature of *Aspa*^*KO*^ mice was lower than *Aspa*^*WT*^ mice in the refed state (Fig. 5e). Feeding also causes a drop in plasma pyrimidines to form a complex endocrine loop with leptin that affects energy balance^24^. Leptin levels were equivalent in the fasted state, but refeeding did not recover serum abundance in *Aspa*^*KO*^ mice relative to littermate controls (Fig. 5f).

Finally, we explored protein expression changes in WAT from *Aspa*^*WT*^ and *Aspa*^*KO*^ mice during fasting and refeeding. ASPA expression greatly increased upon refeeding (Fig. 5g), indicating NAA consumption, and thus decreased CAD activity. Indeed, we saw greater phospho-CAD and total CAD signal in the *Aspa*^*KO*^ mice compared to *Aspa*^*WT*^ in the refed state, suggesting CAD and *de novo* pyrimidine synthesis remain unopposed when NAA levels are high. These data suggest NAA and ASPA regulate CAD, and *Aspa* deletion does not permit lower postprandial pyrimidine synthesis for body temperature homeostasis.

### Adipocyte-specific *Aspa* deletion sufficiently accumulates NAA to lower postprandial body temperature.

**Extended Fig 3 |:**
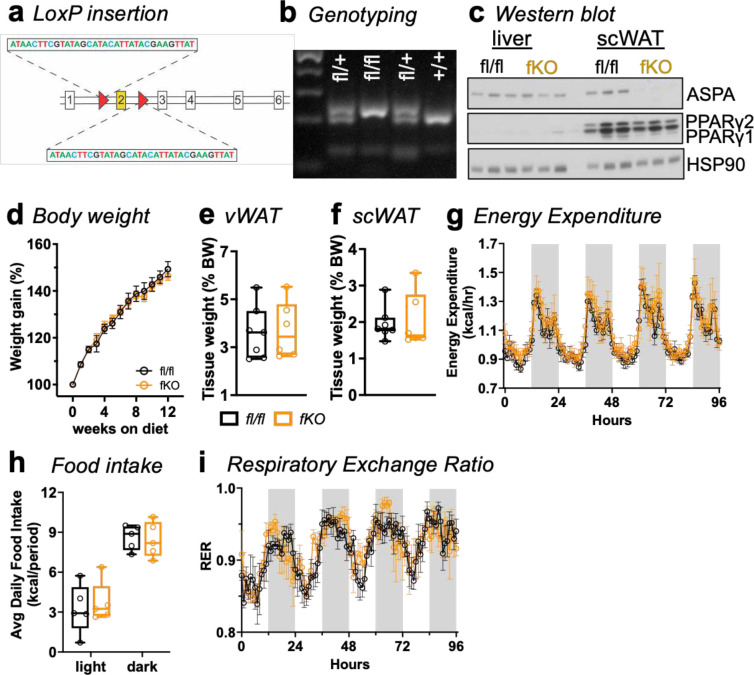
Generation of the mouse model for conditional *Aspa* gene deletion. **a,** *Aspa*^*fl/fl*^ mice were generated using CRISPR/Cas9 gene editing. Exon 2 of the *Aspa* gene was targeted by sgRNAs designed complementary to intronic sequences flanking the exon, followed by insertion of loxP sequences by DNA donor oligonucleotides. **b,** Validation of floxed allele by genotyping. PCR analyses of floxed alleles at the targeted loci in genomic DNA extracted from ear clips of wild-type (+/+), fl/+, and fl/fl mice. PCR products were run on agarose gels with expected band sizes: wild-type (+) 217 bp and loxP allele (fl) 251 bp. **c,** Immunoblot validation of adipocyte-specific *Aspa* deletion in scWAT vs liver, probed for ASPA, PPARγ, and HSP90 (loading control). **d,** Body weight measurements of *Aspa*^*fl/fl*^ and *Aspa*^*fKO*^ mice fed NCD over 12-week period starting at 6 weeks of age; shown as percent weight gain (n=7,10 mice/group). Data represent mean ± s.e.m. No significant difference across genotypes as determined by mixed-effects model. **e,** vWAT and **(f)** scWAT depot tissue weights, shown as percentage of body weight (n=7,10 mice/group). For statistical analyses, performed unpaired two-tailed Student’s *t*-test. Mice were individually housed and monitored in CLAMS cages during a 96 h period with measurements of: **(g)** Energy expenditure (EE); **(h)** Food intake (n=5 mice/group); **(i)** Respiratory exchange ratio (RER). Statistical analyses of **(g-i)** were performed by ANCOVA with lean body mass as a covariate. **e-f,h,** Data represented as box-and-whisker plots using the Min-to-Max method in GraphPad Prism: box limits, 25^th^ to 75^th^ percentiles; center line, median; whiskers, minimum and maximum values.

In contrast to Canavan disease, *Aspa*^*KO*^ mice do not exhibit demonstrably shortened lifespan, but fail to grow and develop macrocephaly^13,25^. The whole-body knockout may influence metabolic and behavioral phenotypes due to *Aspa* loss in other tissues, such as the brain. To determine the adipocyte-specific role of NAA and ASPA in adipose tissue and whole-body energy balance, we used CRISPR/Cas9 to insert loxP sites flanking exon 2 by homology-directed repair for generation of conditional *Aspa* knockout alleles (*Aspa*^*fl/fl*^, Extended Data Fig. 3a). The presence of loxP sites in the targeted regions was confirmed by Sanger sequencing and genotyping PCR of founder floxed alleles (Extended Data Fig. 3b). To examine the adipocyte-specific impact of ASPA on energy balance, we crossed *Aspa*^*fl/fl*^ mice with mice expressing Cre recombinase under control of the adiponectin promoter (*Adipoq-Cre)* to generate *Aspa* knockout in mature fat cells. Adipocyte-specific deletion of *Aspa* in the *Aspa*^*fl/fl*^*:Adipoq-Cre* was validated by Western blot. *Aspa* was successfully knocked out in the WAT of *Aspa*^*fl/fl*^*:Adipoq-Cre* (*Aspa*^*fKO*^) mice but not in the *Aspa*^*fl/fl*^ littermates or in the liver of either genotype (Extended Data Fig. 3c).

**Fig. 6 |:**
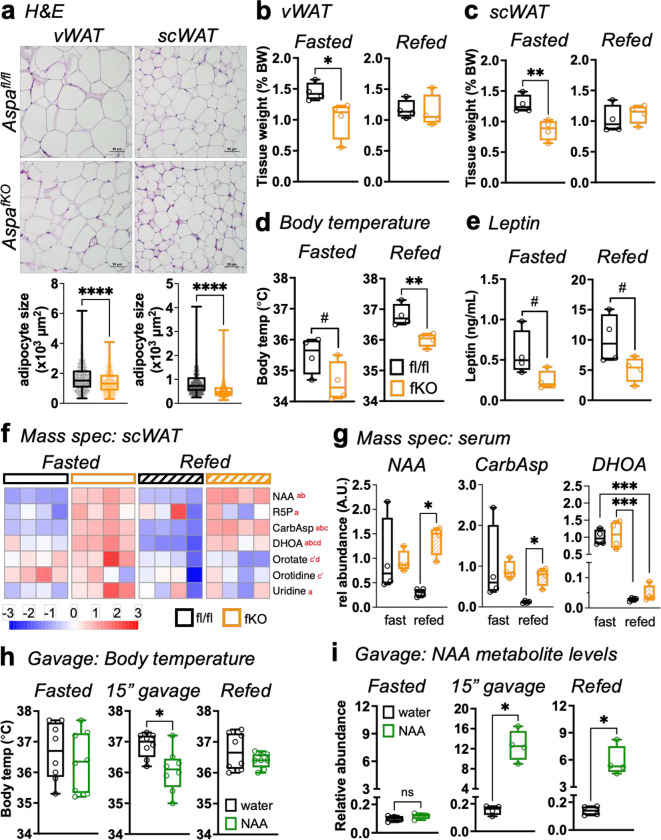
NAA from adipose tissue performs post-prandial body temperature regulation. **a,** Representative histological analysis by H&E and mean adipocyte size (μm^2^) of vWAT and scWAT across 3–4 fields of view (n=3–4 mice/group); scale bars, 50μm. Necropsy data from fasted and refed mice: **(b)** vWAT and **(c)** scWAT, shown as % of body weight (n=4 mice/group/condition). Body temperature measurements and serum of *Aspa*^*fl/fl*^ and *Aspa*^*fKO*^ mice were taken following 16 h fast and following 6 h of refeeding: **(d)** body temperature, **(e)** serum leptin levels (n=4 mice/group). **f,** Relative abundance of NAA and pyrimidine metabolites in scWAT in fasted and refed states. Shown as z-score of log_2_ transformed values. a (fasted fl vs fKO), b (refed fl vs fKO), c (fl fasted vs refed), d (fKO fasted vs refed). abcd *P<*0.05, a’b’c’d’ *P*<0.1. **g,** Relative abundance of NAA and pyrimidine metabolites in serum of *Aspa*^*fl/fl*^ and *Aspa*^*fKO*^ mice in fasted and refed states. **h,** Body temperature measurements of WT mice following 16 h fast, gavage, and after one h of refeeding (n=8/group). **i,** Relative abundance of NAA in serum of WT mice from **(h).** **(f,g,i)** measured by IC-MS analysis (n=4 mice/group/condition). **(a-e,g-i)** Data represented as box-and-whisker plots using the Min-to-Max method in GraphPad Prism: box limits, 25^th^ to 75^th^ percentiles; center line, median; whiskers, minimum and maximum values. ^#^*P*<0.1, *P<0.05, **P<0.01, ***P<0.001, ****P<0.0001 by unpaired two-tailed Student’s *t*-test.

Upon confirming adipocyte-specific *Aspa* deletion, we assessed energy balance parameters of ad-libitum *Aspa*^*fKO*^ and littermate *Aspa*^*fl/fl*^ controls. Contrary to our expectations, weight gain (Extended Data Fig. 3d) and WAT mass (Extended Data Fig. 3e,f) were similar. *Aspa*^*fKO*^ and littermate *Aspa*^*fl/fl*^ controls also displayed indistinguishable energy balance profiles (Extended Data Fig. 3g,h,i). Based on the *Aspa*^*KO*^ studies, we expected that adipocyte-specific deletion of *Aspa* may harbor smaller fat cells in WAT. To examine this notion, we performed quantitative image-based histological analysis to measure adipocyte hyperplasia in *Aspa*^*fKO*^ WAT compared to controls. We observed adipocytes from *Aspa*^*fKO*^ WAT were significantly smaller (Fig. 6a) resembling *Aspa*^*KO*^ mice and favoring healthy expansion of WAT depots^26,27^.

We then performed fasting/refeeding experiments to determine if adipocyte-specific *Aspa* deletion recapitulated the lower postprandial body temperature phenotype observed in *Aspa*^*KO*^. In the *Aspa*^*fKO*^ model, WAT depots weighed less than those of *Aspa*^*fl/fl*^ littermate controls in the fasted state (Fig. 6b,c). Body temperature of *Aspa*^*fKO*^ mice trended lower in the fasted state and was significantly lower than *Aspa*^*fl/fl*^ in the refed state, suggesting adipose tissue-derived NAA and ASPA are important in the regulation of body temperature (Fig. 6d). We also measured leptin levels in these mice and observed decreased leptin in the *Aspa*^*fKO*^ mice (Fig. 6e). Because these mice were similar in body weight (Extended Data Fig. 3d), the lower leptin levels suggest a role for NAA and ASPA in regulation of leptin, which has been linked to regulation of postprandial body temperature^24,28^.

Mice with global *Aspa* loss produced systemic NAA and elevated plasma levels of pyrimidines and their intermediates. Given that fat cells express the machinery for secretion of NAA, we hypothesized *Aspa*^*fKO*^ contributed to the accumulation of pyrimidines that lowered postprandial body temperature. To analyze how nutrient state affects metabolites across the two genotypes, we performed mass spectrometry analysis of scWAT and serum from *Aspa*^*fKO*^ and littermate controls. NAA levels were higher in scWAT of *Aspa*^*fKO*^ mice in the fasted and refed states (Fig. 6f). Similarly, CarbAsp and DHOA were also higher in *Aspa*^*fKO*^ in both conditions. Other metabolites, such as R5P and uridine were higher in *Aspa*^*fKO*^ only in the fasted state. Downstream pyrimidines orotate and orotidine were not different across genotypes, but differed across nutrient conditions, with higher levels in the fasted state. When we analyzed serum metabolite levels in these mice, we found that adipocyte-specific *Aspa* deletion was sufficient to raise NAA levels systemically in *Aspa*^*fKO*^ mice (Fig. 6g), predominantly in the refed state when ASPA expression increases in *Aspa*^*fl/fl*^ but remains low in *Aspa*^*fKO*^ mice. Consequently, CarbAsp also remained higher in the *Aspa*^*fKO*^ in the refed state, while *Aspa*^*fl/fl*^ mice had significantly lower levels in the refed state.

Upon discovering that adipocyte-specific *Aspa* deletion sufficiently raised systemic NAA levels, lowered postprandial body temperature, and increased the abundance of *de novo* pyrimidines, we asked whether acute NAA treatment in wild-type (WT) mice was sufficient to induce similar changes in body temperature. WT mice were fasted overnight for 16 h, followed by baseline rectal temperature measurements. We then gavaged mice with pH-balanced water or NAA and measured rectal temperature 15 min post-gavage. Finally, mice were refed for one h followed by another round of body temperature measurements. We found that acute treatment with NAA (500 mg/kg BW) was sufficient to induce a significant decrease in body temperature in WT mice 15 min post-gavage, while there was no difference in body temperature in fasted or refed states (Fig. 6h). To confirm that NAA gavage sufficiently elevated serum NAA levels, we performed targeted mass spectrometry analysis of serum samples from the three timepoints. We found that NAA gavage increased serum NAA levels nearly 100-fold, and serum levels decreased significantly after refeeding, though remained elevated compared to water-treated mice (Fig. 6i). These results suggest NAA impacts pyrimidine synthesis to regulate body temperature by maintaining elevated levels of CarbAsp and DHOA from greater adipose tissue CAD activity. Moreover, acute NAA elevation is sufficient to decrease body temperature in fasted mice.

## Discussion

The brain-enriched amino acid NAA carries acetate for the synthesis of acetyl-CoA and myelin lipids in oligodendrocytes. Thus, the presence of NAA synthesis and hydrolysis machinery in fat cells predicts ASPA provides lipogenic acetate for adipose tissue expansion. Instead, we present new evidence demonstrating NAA generated from adipose tissues can lower postprandial body temperature. Our observations identify endocrine roles for adipose tissue-derived NAA that enable metabolic flux into CAD to synthesize pyrimidine pools that control body temperature. These findings align with the impact of the pyrimidine uridine from adipose tissue, which reduces body temperature in fasted mice^24^.

Disruption of NAA hydrolysis by constitutive *Aspa* deletion causes systemic effects that promote metabolic flexibility. *Aspa*^*KO*^ show greater amplitude in RER during light-to-dark transitions. These observations suggest *Aspa* deletion and hyper-elevation of NAA enhance fat catabolism in the fasted state and lipogenesis in the fed state. Previous work established intraperitoneal injections of uridine increased RER and carbohydrate consumption^24^, and our current study identifies that whole-body NAA elevation contributes to pyrimidine synthesis and lowered energy demands. The recent demonstration that uridine supplies ribose for glycolysis and gluconeogenesis^29,30^ suggests NAA levels may serve as a critical signal in cells during long-term starvation to pursue alternative substrates for metabolic homeostasis.

Nucleotides are necessary for DNA and RNA synthesis, with greater demand in highly proliferative cells. Thus, the levels of purine and pyrimidine nucleotides within a cell are tightly regulated and maintained at equal ratios and optimal concentrations to meet cellular demands. Nucleotide imbalance results in the activation of replication stress machinery and disruption of cell growth and proliferation^31^. Our metabolic tracing efforts suggest NAA accumulation in fat cells increased de novo pyrimidine synthesis and this metabolic outcome is likely maintained *in vivo*. Nucleotide biosynthesis is frequently overlooked in adipocytes as they are terminally differentiated cells and do not re-enter proliferation. However, fat cells produce uridine in the fasted state, and our results are aligned with increasing evidence that CAD activity and *de novo* pyrimidine synthesis contribute to energy balance in mice and humans^24,32,33^.

CAD is the initial and rate-limiting enzyme for de novo pyrimidine synthesis. Integrated metabolomic and proteomic studies demonstrated that high NAA levels unveil CAD activity to increase the abundance of pyrimidines. Because NAA accumulation cannot be shut off by ASPA upon refeeding in *Aspa* knockout models, *de novo* pyrimidine levels remained unopposed and postprandial body temperature did not achieve levels observed in littermate controls. Due to structural similarities with n-acetylglutamine, NAA is able to activate carbamoyl phosphate synthetase I (CPSI)^34^. Interestingly, CPSI shares a single mammalian homolog named carbamoyl phosphate synthetase II (CPSII), which is expressed as one of the enzymatic domains of the large CAD protein complex and shares 70% sequence similarity with CPSI. However, CPSII does not bind N-acetylglutamate. We speculate that NAA acts as an allosteric activator of CAD in the fasted state for the synthesis of pyrimidines in adipose tissue. It will now be important to determine how nutrient sensing in WAT governs NAA interactions with CAD for energy balance regulation.

WAT is highly innervated by local sensory nerve fibers and sympathetic nerve fibers that relay nutrient availability to the central nervous system^35,36^. Through this mechanism, the adipocyte-specific and pyrexic hormone leptin acts on nerve fibers to raise postprandial body temperature and thermic responses to refeeding^24,28^. In future studies, we expect the decreased body temperature observed in *Aspa* knockout mouse models from higher circulating NAA in the refed state converges leptin and pyrimidine action within sensory fibers to govern nutrient-sensing for physiological homeostasis.

Limitations of our study include the fact that we do not rule out the possibility that NAA synthesis and disposal occurs elsewhere to regulate the whole-body pyrimidine pool. N-acetylated amino acids undergo glomerular filtration and reabsorption in the proximal tubule where they are hydrolyzed by aminoacylase 1^37^. ASPA expression also occurs in the kidney, though its role there remains uncharacterized. Further studies using our newly developed *Aspa*^*fl/fl*^ mice may shed light on other tissue-specific responses and targets of circulating NAA. Furthermore, although we identified NAA from WAT blunts postprandial body temperature, we have not identified the precise signal integration site in the CNS. Finally, our data were primarily collected in mice, but the lower serum NAA levels in obesity and lessened postprandial body temperature responses in this population^28^ suggest that this system is also operative in humans. Our findings justify future studies in humans to examine if dietary interventions that raise NAA influence body weight.

## Methods

### Animal Studies

All animal procedures were approved by the Institutional Animal Care and Use Committee of Baylor College of Medicine (Animal Protocol AN-6411). Experimental animals received humane care according to criteria in the “Guide for the Care and Use of Laboratory Animals” (8^th^ edition, revised 2011). Experimental animals were housed (no more than four per cage) in a barrier-specific pathogen-free animal facility with 12 h dark-light cycle and free access to water and normal chow (Harlan Laboratories 2920X), unless otherwise specified. We used the C57BL/6N *Aspa*^*tm1b(EUCOMM)Wtsi*^ mouse strain with deletion of *Aspa* exon 2. Mice that were homozygous for the *Aspa* allele (*Aspa*^*KO*^) have a total knockout of the gene. Experiments were conducted using littermate-controlled mice maintained on a C57BL/6J background. At the end of each experiment, mice were euthanized by cervical dislocation while under isoflurane anesthesia. After euthanasia, tissues were collected and either fixed in 10% formalin or flash-frozen in liquid N_2_ and stored at −80 °C until use. All experiments adhered to ARRIVE Guidelines.

### In vitro studies.

Stromal vascular fraction (SVF) cells were isolated from mouse subcutaneous WAT. Fat depots were digested in PBS containing collagenase I (1.5 U/ml; Roche #17100–017) and dispase II (2.4 U/ml; Sigma #D4693) supplemented with 10 mM CaCl_2_ at 37 °C for 45–50 min. The primary cells were filtered twice through 70 μm cell strainers and centrifuged at 700 rcf to collect the SVF. The SVF cell pellets were rinsed and plated. Adipocyte differentiation was induced by treating confluent cells in DMEM/F12 medium containing Glutamax (ThermoFisher #10565–018), 10% dialyzed FBS (Gibco #A3382001), with 0.250 mM isobutylmethylxanthine (Sigma #13347), 1 mM rosiglitazone (Cayman Chemical Co. #71740), 1 mM dexamethasone (Tocris Biosciences #1126), 850 nM insulin (Sigma I5500), and 1 nM T3 (Sigma #T-074). Three days after induction, cells were switched to the maintenance medium containing 10% dialyzed FBS, 1 mM rosiglitazone, 1 mM dexamethasone, 850 nM insulin, 1 nM T3. Experiments occurred 8–10 days after induction of differentiation, and Oil-Red-O (ORO, Biovision K580) was used to assess overall lipid accumulation. Subcutaneous human preadipocytes (Zen-Bio) were differentiated as described above.

### Antibodies and Western blotting.

Tissue and whole cell lysates were extracted using Protein Extraction Reagent (Thermo Fisher) supplemented with Halt Protease and Phosphatase Inhibitor Cocktail (Thermo Fisher). Immunoblotting was performed with lysates run on 4–12% Bis-Tris NuPage gels (Life Technologies) and transferred onto Immobilon-P Transfer Membranes (Millipore) followed by antibody incubation. Immunoreactive bands were visualized by chemiluminescence. The following antibodies were used for immunoblotting: α-ASPA (Abcam #154503), α-ADIPOQ (GeneTex #GTX112777), α-PPARG (Cell Signaling #2443), α-UCP1 (Abcam #ab10983), α-phospho-CAD S1859 (Cell Signaling #12662), α-CAD (Cell Signaling #11933), α-phospho-P70S6 Kinase T389 (Cell Signaling #9205), α-P70S6 Kinase (Cell Signaling #2708), α-DHODH (Proteintech #14877-I-AP), α-HSP90 (Cell Signaling #4877).

### RNA isolation and qPCR.

Total RNA was extracted using the RNeasy Mini Plus kit (*in vitro* cells) or RNeasy Lipid Tissue kit (tissues) (Qiagen). cDNA was synthesized from total RNA using qScript (QuantBio #95048–100). Relative mRNA expression was measured with SsoAdvanced Universal Probes Supermix reactions (Bio-Rad #175284) read out with a QuantStudio 3 real-time PCR system (Applied Biosystems). TATA-box binding protein (Tbp) was the invariant control. Roche Universal Probe Gene Expression Assays (mouse) or individual TaqMan Gene Expression Assays (Thermo Fisher) were used as previously described^38^.

### Reverse Phase Protein Arrays.

Protein lysates were prepared by the BCM Antibody-Based Proteomics Core for reverse phase protein array assays. The Aushon 2470 Arrayer (Aushon BioSystems) with a 40 pin (185 μm) configuration was used to spot lysates onto nitrocellulose-coated slides (Grace Bio-Labs). The slides were probed with >220 antibodies against total and phospho-proteins using an automated slide stainer (Dako). Primary antibody binding was detected using a biotinylated secondary antibody followed by streptavidin-conjugated IRDye 680 fluorophore (LI-COR). Fluorescent-labeled slides were scanned on a GenePix AL4200 and the images were analyzed with GenePix Pro 7.0 (Molecular Devices). Background-subtracted total fluorescence intensities of each spot were normalized for variation in total protein (Sypro Ruby) and nonspecific labeling.

### Generation of a conditional *Aspa* allele.

*Aspa*^*fl/fl*^ mice were generated by the Genetically Engineered Rodent Models Core at BCM using previously established methods^39^. We employed Cas9-initiated homology-directed repair (HDR) using a pair of single guide RNAs (sgRNAs) coupled with a long single-stranded oligodeoxynucleotide donor (lssDNA) template harboring short homology arms and loxP-flanked exons. The sgRNAs and the lssDNA donor were used to insert loxP sites 5’ and 3’ of exon 2 of the mouse *Aspa* gene. To minimize the probability of off-target events, only sgRNAs predicted to have off-target sites with three mismatches or more were used to target Cas9 endonuclease activity to intronic sequences flanking exon 2. Two hundred C57BL/6J pronuclear-stage zygotes were co-injected with Cas9 mRNA, sgRNAs, and lssDNA^39^. Following micro-injection, zygotes were transferred into pseudopregnant ICR recipient females at approximately 25–32 zygotes per recipient. Sanger sequencing of cloned loxP sites and founder line genotyping from mouse genomic DNA confirmed loxP insertions and sequence fidelity. Progeny generated from putative founders were also sequence-confirmed for the fidelity of the loxP sequences and floxed exons. *Aspa*^*fl/fl*^ mice were crossed to Adipoq-Cre (Jackson Laboratory #028020) to generate adipocyte-specific *Aspa* knockout (*Aspa*^*fKO*^) and littermate controls (*Aspa*^*fl/fl*^). *Aspa*^*fl/fl*^ mice are distributed upon request.

### Genotyping.

DNA was extracted from mouse ear clips and used in PCR reactions with primers designed to detect the WT (5’- TGTCCCTGATCCAGTAGTCAT, 3’- GAGTTAGTTCTAGGACAGCAGT, 426bp band) and mutant (Lar3) (5’- same as WT, 3’- CAACGGGTTCTTCTGTTAGTCC, 388bp band) sequences in *Aspa* whole-body knockout mice. Primers were used to detect the 5’ loxP sequences (forward: CAGTGCACCATAACTACAAGCA, reverse: TCTGAAACCTCTGCAAATGACA) in the conditional mice. The PCR product was run on agarose gels. Both whole-body knockout and Cre transgenic mice were genotyped according to protocols provided by the Jackson Laboratory.

### Indirect calorimetry and body temperature measurements.

Mice were maintained on experimental diets and housed at room temperature in Comprehensive Laboratory Animal Monitoring Systems Home Cages (CLAMS-HC, Columbus Instruments). Oxygen consumption, CO_2_ emission, energy expenditure, food and water intake, and activity were measured for six days (BCM Mouse Metabolism and Phenotyping Core). Mouse body weight was measured, and body composition was examined by magnetic resonance imaging (Echo Medical Systems) prior to indirect calorimetry. The body temperature was measured by rectal probe connected to a digital thermometer (BAT-12 Microprobe-Thermometer; Physitemp; NJ, USA). All treatment and temperature measurements were performed at ambient room temperature (23–25 °C). Statistical analysis of energy expenditure was performed by ANCOVA with lean body mass as a covariate using the CalR web-based tool^17^.

### Glucose and insulin tolerance tests.

Mice were maintained on experimental diets for 10–18 weeks then subjected to tolerance tests. To assess glucose tolerance, mice were fasted for 16 h and glucose was administered (1.5 g/kg body weight) by intraperitoneal (IP) injection. To assess insulin tolerance, mice were fasted 4 h prior to insulin IP injection (1.5 U/kg body weight). Blood glucose levels were measured by handheld glucometer. Serum was collected after fasting and during glucose tolerance tests for insulin quantification.

### ELISAs and FFA assays.

Serum collected from fasted, re-fed, and/or ad-libitum fed mice was used to measure insulin (Millipore #EZRMI-13K), leptin (Crystal Chem #90030), adiponectin (Thermo Fisher #KMP0041), and free fatty acids (ZenBio #sfa-1) as noted.

### Histology.

Formalin-fixed, paraffin-embedded adipose sections were stained with hematoxylin and eosin (H&E) by the BCM Human Tissue Acquisition and Pathology Core. Images were captured (20X) using a Nikon Ci-L Brightfield microscope and adipocyte morphometry was quantified using ImageJ Adiposoft software^40^.

### Fluorescence microscopy.

Human adipocytes were fixed in 4% paraformaldehyde. Glycine was used to quench auto-fluorescence derived from residual paraformaldehyde. Fixed cells were incubated with rabbit a-ASPA (Abcam #154503) primary antibody at 4 °C overnight in 2% BSA/0.1% saponin/PBS, washed in 1x PBS three times (5 min/wash), and then incubated with Alexa Fluor 647-conjugated goat α-rabbit IgG (Life Technologies). DAPI (Sigma #D8417) and LipidTOX (Life Technologies #H34475) were used for nuclei and lipid droplet labeling, respectively. Imaging was performed with the Olympus IX83 epifluorescence deconvolution microscope (Olympus).

### Metabolomics.

Targeted measurements of metabolites in 3T3-L1 cells were carried out by the BCM Dan L Duncan Cancer Center CPRIT Cancer Proteomics and Metabolomics Core using previously described methods^41^. To determine the relative abundance of metabolites in tissues, extracts were prepared and analyzed by high resolution mass spectrometry. Briefly, approximately 50 mg of individual tissue samples were homogenized with Precellys Tissue Homogenizer. Metabolites were extracted using ice-cold 0.1% trichloroacetic acid in 90/10 (v/v) Acetonitrile/water. Then, extracts were centrifuged at 17,000 g for 5 minutes at 4 °C, and supernatants were transferred to clean auto sampler vials. In the end, 10 μL volume of each sample was injected for analysis by liquid chromatography-mass spectrometry (LC-MS). Thermo Vanquish LC system included a SeQuant Zic-cHilic column (3 μm particle size, 100 × 2.1 mm) with the column compartment kept at 30 °C. Mobile phase A (MPA; weak) was 95/5 (v/v) acetonitrile/(200 mM ammonium acetate buffer, pH 5.8), and mobile phase B (MPB; strong) was 50/45/5 acetonitrile/water/(200 mM ammonium acetate buffer, pH 5.8). The autosampler tray was chilled to 4 °C. The mobile phase flow rate was 300 μL/min, and the gradient elution program was: 0–2 minutes, 20% MPB; 2–6 minutes, 20–80% MPB; 6–10 minutes, 80% MPB; 10–11 minutes, 80–20% MPB. The total run time was 15 minutes. Data were acquired using a Thermo Orbitrap Fusion Tribrid Mass Spectrometer under the ESI positive ionization mode at a resolution of 240,000. Raw data files were imported to Thermo Trace Finder software for final analysis. The relative abundance of each metabolite was normalized by tissue weight.

For the ex vivo adipose tissue studies, fat pads were excised and cut into ~50 mg pieces in PBS on ice. At least two tissue pieces per mouse were allocated to each treatment group. Each tissue was placed in a well of a 24-well plate containing 0.5 ml DMEM/F12. After overnight incubation at 37 °C, 5% CO_2_, tissues were snap-frozen in liquid N_2_ and media snap-frozen on dry ice. Polar metabolites were extracted as previously described^42^. Tissue samples were first pulverized, then 10 to 30 mg tissue was weighed and used for extraction with 1 ml of ice-cold 80% methanol/water. Samples were homogenized in TissueLyser II and incubated on ice for 10 min, then centrifuged at 20,000 × g at 4 °C for 10 min. Supernatant was collected and dried in vacuum concentrator and then stored in −80 °C until reconstitution for metabolomics analysis. For media samples, 10 uL was collected for extraction with 500 uL of ice-cold 80% methanol/water. Samples were vortexed and centrifuged at 20,000 × g for 10 min at 4 °C. Supernatant was collected and dried in vacuum concentrator and then stored in −80 °C. All dried samples were reconstituted into 30 μL of sample solvent (water:methanol:acetonitrile, 2:1:1, v/v/v) and were centrifuged at 20,000 × g at 4 °C for 3 min. The supernatant was transferred to LC vials for the analysis.

Chromatography separations were carried out using a HILIC with an Xbridge amide column (100 X 2.1 mm internal diameter [i.d.], 3.5 μm; Waters) on the Vanquish Horizon UHPLC system. The column temperature maintained at 40 °C, autosampler at 4 °C, and injection volume of 3 μL. The column was employed with mobile phase A: 5 mM ammonium acetate in water (pH=9.0 with addition of ammonium hydroxide), and mobile phase B: 100% acetonitrile. Linear gradient was: 0 min, 85% B; 1.5 min, 85% B; 5.5 min, 35% B; 10.5 min, 35% B; 10.6 min, 10% B; 14 min, 10% B; 14.5 min, 85% B, and 24 min, 85% B. Flow rate was 0.3 mL/min. The mass spectrometry analysis was performed on an Orbitrap Exploris 480 mass spectrometer equipped with a heated electrospray ionization (HESI) probe. For polar metabolites, the relevant parameters were listed: heater temperature, 120 °C; sheath gas, 30; auxiliary gas, 10; sweep gas, 3; spray voltage, 3.6 kV for positive mode and 2.5 kV for negative mode. Capillary temperature was set at 320 °C, and S-lens was 55. A full scan range was set at 60–900 (mass to charge [m/z]). The resolution was set at 60000 (at m/z 200). Customized mass calibration was performed before data acquisition using Xcalibur. Metabolite identification and peak integration were carried out using Skyline software (v23.1.0). The integrated peak intensity was used for further data analysis.

### U-^13^C glucose flux and IC-MS analysis.

The SVF-derived fibroblasts were induced to mature white adipocytes following the standard protocol described above. Mature white adipocytes were switched to glucose-free, pyruvate-free DMEM (Gibco) containing 10% dialyzed FBS. U-^13^C6 glucose (Cambridge Isotope Laboratories, Inc, CLM-6750-PK) was added to media at final concentration of 11.1 mM for 24 h, unless otherwise noted. After 24 h of incubation, medium was aspirated, and cells were washed twice with ice-cold PBS followed by liquid nitrogen.

To determine the incorporation of ^13^C_6_-glucose carbon into intracellular intermediates, extracts were prepared and analyzed by high-resolution mass spectrometry. Metabolites were extracted in 1 ml ice-cold 80/20 (v/v) methanol/water containing 0.1% ammonium hydroxide. Samples were then vortexed for 2 minutes, centrifuged at 17,000 × *g* for 5 minutes at 4 °C, and supernatants were transferred to clean tubes and evaporated to dryness under nitrogen. Samples were reconstituted in deionized water, then 10 μl were injected into a Thermo Scientific Dionex ICS-5000+ capillary ion chromatography (IC) system containing a Thermo IonPac AS11 250×2 mm 4 μm column. IC flow rate was 360 μl/min (at 30 °C), and the gradient conditions were as follows: started with an initial 1 mM KOH, increased to 35 mM at 25 min, then to 99 mM at 39 min, and maintained at 99 mM for 10 min. The total run time was 50 min. To assist desolvation and increase sensitivity, methanol was delivered by an external pump and combined with the eluent via a low dead volume mixing tee.

Data were acquired using a Thermo Orbitrap Fusion Tribrid Mass Spectrometer under negative electrospray ionization (ESI) mode. The heated electrospray ionization (H-ESI) source and global MS parameters for the acquisition were as follows: scan range = 80–800 m/z; multiplex ions = false; isolation mode = quadrupole; detector type = Orbitrap; Orbitrap resolution = 240,000 (at m/z 200); RF lens (%) = 50; AGC Target = 200,000; injection ions for all available parallelizable time = true; maximum injection time (ms) = 100; microscans = 1; data type = profile; polarity = negative; source fragmentation = disabled; use EASY-IC = true; include start and end times = false; scan cycle time = 3 seconds. The raw data files were imported to Thermo Trace Finder software for final analysis. For ^13^C isotopolog analysis, ElemCor software was used for natural abundance correction and enrichment calculation^43^. The relative abundance of each isotopolog was calculated as the isotopolog area divided by (normalized by) the sum of all isotopolog peak areas.

### ARIC data.

Data from the ARIC (Atherosclerosis Risk In Communities) Study visit 5 were downloaded to explore the interactions between BMI and metabolites in serum from humans^44^. A Linear regression model was applied to evaluate interactions between BMI and metabolites vary with gender (F/M), race (B/W), and age (continuous) adjusted in the model. The significance threshold was calculated by 0.05 divided by the number of tests to adjust for multiple comparisons. Interactions with p-value less than the threshold were reported as significant. R program 4.3.1 was utilized for the statistical analysis.

### Statistical analysis and reproducibility.

All measurements were taken from distinct biological samples. Unless otherwise noted, all statistical analyses were performed using GraphPad Prism (version 9) and tests described in the figure legends. All data are presented as mean ± standard error of the mean (SEM), unless otherwise noted. Statistical significance shown as ^#^ p < 0.1, * p < 0.05, ** p < 0.01, *** p < 0.001, *** p < 0.0001.

## Extended Data

**Extended Data 1 |:**
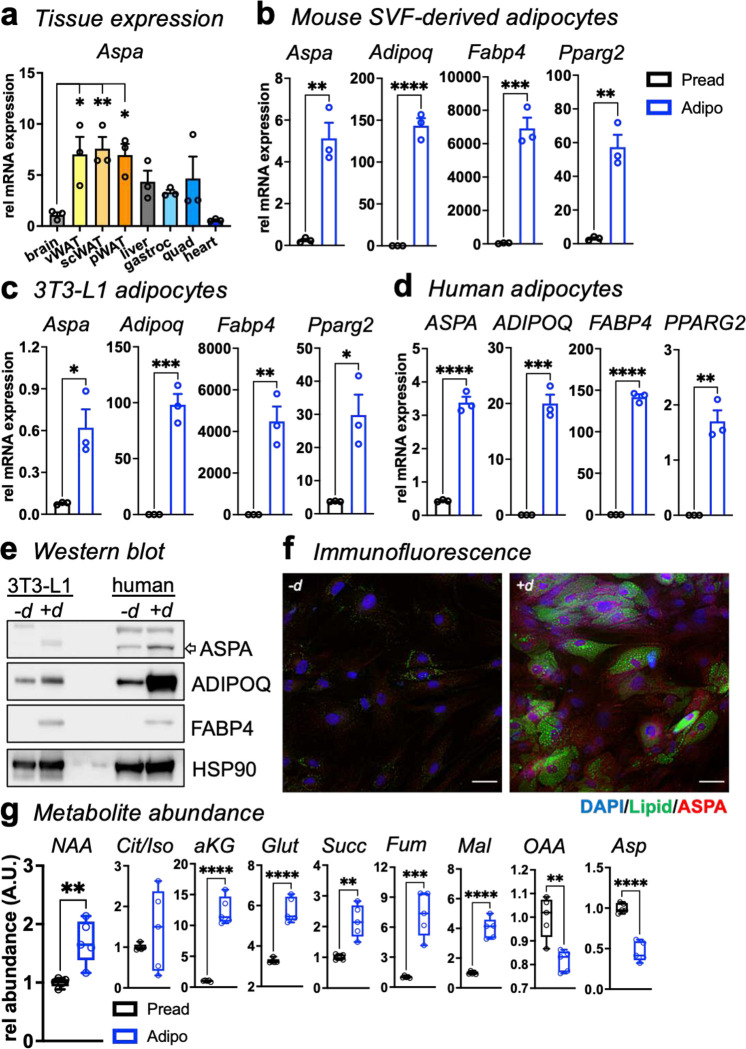
*Aspa* is highly expressed in differentiated adipocytes and adipose tissue. **a**, *Aspa* gene expression across mouse tissues (n=3 biological replicates). Data represent mean ± s.e.m. *P<0.05, **P<0.01, ****P<0.0001 by ordinary one-way ANOVA followed by Dunnett’s multiple comparisons test of brain vs different tissues. Expression of *Aspa* and adipogenic genes (*Adipoq, Fabp4,* and *Pparg2*) in pre-adipocytes (Pread) and mature adipocytes (Adipo) of mouse: **(b)** SVF-derived adipocytes, **(c)** 3T3-L1 adipocytes. Data represent mean ± s.e.m. **d,** Expression of *ASPA* and adipogenic genes (*ADIPOQ, FABP4*, and *PPARG2*) in human primary pre-adipocytes (Pread) and mature adipocytes (Adipo) (n=3 replicates/group). Data represent mean ± s.e.m. **e,** Immunoblot of 3T3-L1 cells and human primary cells probed for ASPA, ADIPOQ, and PPAR*γ* before (−d) and after adipocyte differentiation (+d). **f,** Immunofluorescent staining of ASPA (red) and co-labeling of lipid droplets (green, LipidTox), and nuclei (blue, DAPI) of primary human adipocytes before and after adipocyte differentiation. **g,** Relative abundance of NAA and TCA metabolites in 3T3-L1 pre-adipocytes (Pread) and mature adipocytes (Adipo) (n=5 replicates/group). Data represented as box-and-whisker plots using the Min-to-Max method in GraphPad Prism: box limits, 25^th^ to 75^th^ percentiles; center line, median; whiskers, minimum and maximum values. **(b-d,g)** *P<0.05, **P<0.01, ***P<0.001, ****P<0.0001 by unpaired two-tailed Student’s *t*-test.

**Extended Data 2 |:**
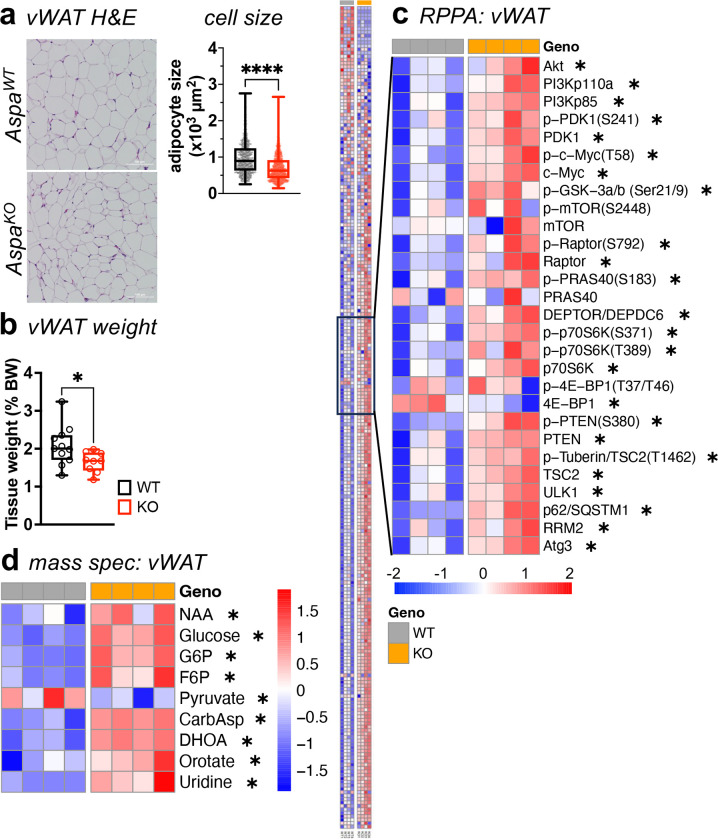
Smaller adipocytes and greater production of pyrimidines resulting from *Aspa* deletion also seen in vWAT. *Aspa*^*WT*^ and *Aspa*^*KO*^ mice were fed NCD for 12 weeks. **a,** Representative histological analysis by H&E and mean adipocyte size (μm^2^) of vWAT across 3–5 fields of view (n=4 mice/group); scale bars, 100μm. **b,** vWAT depot tissue weight, shown as percentage of body weight (n=11,9 mice/group). **a-b,** Data represented as box-and-whisker plots using the Min-to-Max method in GraphPad Prism: box limits, 25^th^ to 75^th^ percentiles; center line, median; whiskers, minimum and maximum values. **c,** Reverse-phase protein array (RPPA) analysis of vWAT from *Aspa*^*WT*^ and *Aspa*^*KO*^ mice. **d,** Heat map of relative abundance of NAA, glycolytic, and pyrimidine metabolites in vWAT of *Aspa*^*WT*^ and *Aspa*^*KO*^ mice, measured by IC/MS targeted analysis. **c-d,** Data shown as z-score of log_2_ transformed values (n=4 mice/group). **a-d,** ^*#*^*P*<0.1, *P<0.05, ****P<0.0001 by unpaired two-tailed Student’s *t*-test.

## Data Availability

The authors declare that other source data supporting the findings of this study within the article and its Supplementary Information files are available upon reasonable request to the corresponding author. Source data are provided with this paper.
